# Metabolomic profiling of metoprolol hypertension treatment reveals altered gut microbiota-derived urinary metabolites

**DOI:** 10.1186/s40246-020-00260-w

**Published:** 2020-03-11

**Authors:** Chad N. Brocker, Thomas Velenosi, Hania K. Flaten, Glenn McWilliams, Kyle McDaniel, Shelby K. Shelton, Jessica Saben, Kristopher W. Krausz, Frank J. Gonzalez, Andrew A. Monte

**Affiliations:** 1 Laboratory of Metabolism, National Cancer Institute, National Institutes of Health, Bethesda, MD USA; 2 Department of Emergency Medicine & Colorado Center for Personalized Medicine, University of Colorado School of Medicine, Aurora, CO USA; 3 Rocky Mountain Poison & Drug Center, Denver Health and Hospital Authority, Denver, CO USA; 4 Department of Pharmaceutical Sciences, University of Colorado School of Pharmacy, 12401 E 17th Ave, Aurora, CO 80045 USA

**Keywords:** CYP2D6, Hypertension, Lisinopril, Metabolomics, Metoprolol

## Abstract

**Introduction:**

Metoprolol succinate is a long-acting beta-blocker prescribed for the management of hypertension (HTN) and other cardiovascular diseases. Metabolomics, the study of end-stage metabolites of upstream biologic processes, yield insight into mechanisms of drug effectiveness and safety. Our aim was to determine metabolomic profiles associated with metoprolol effectiveness for the treatment of hypertension.

**Methods:**

We performed a prospective pragmatic trial (NCT02293096) that enrolled patients between 30 and 80 years with uncontrolled HTN. Patients were started on metoprolol succinate at a dose based upon systolic blood pressure (SBP). Urine and blood pressure measurements were collected weekly. Individuals with a 10% decline in SBP or heart rate (HR) were considered responsive. Genotype for the *CYP2D6* enzyme, the primary metabolic pathway for metoprolol, was evaluated for each subject. Unbiased metabolomic analyses were performed on urine samples using UPLC-QTOF mass spectrometry.

**Results:**

Urinary metoprolol metabolite ratios are indicative of patient *CYP2D6* genotypes. Patients taking metoprolol had significantly higher urinary levels of many gut microbiota-dependent metabolites including hydroxyhippuric acid, hippuric acid, and methyluric acid. Urinary metoprolol metabolite profiles of normal metabolizer (NM) patients more closely correlate to ultra-rapid metabolizer (UM) patients than NM patients. Metabolites did not predict either 10% SBP or HR decline.

**Conclusion:**

In summary, urinary metabolites predict *CYP2D6* genotype in hypertensive patients taking metoprolol. Metoprolol succinate therapy affects the microbiome-derived metabolites.

## Introduction

Hypertension (HTN) is the most common chronic disease in the USA. Effective treatment remains elusive despite more than 25 drugs approved by the Food & Drug Association (FDA) for its treatment. Only 50% of patients that initiate treatment of their HTN achieve adequate blood pressure (BP) control [1, 2]. This relative ineffectiveness is multifactorial; non-compliance, drug-drug interactions, and non-specific treatment for the polygenic complex disease all contribute to therapeutic failures.

Metabolic profiling may help identify new mechanisms of drug effectiveness and safety amongst patients taking antihypertensives. The Pharmacogenomic Evaluation of Antihypertensive Response (PEAR) study demonstrated that atenolol, a beta-blocker, caused changes in plasma fatty acid levels in Caucasians but not African Americans. These race-dependent changes in ketone body 3-hydroxybutanoic acid and the tricarboxylic acid (TCA) cycle intermediate alpha-ketoglutaric acid demonstrate differential drug effects on upstream biologic processes [3]. Arachidonoyl-carnitine was associated with increased blood glucose amongst these patients. Clearly, atenolol affects numerous metabolic processes in addition to the beta-adrenergic antagonism that is intended for BP control [4]. Alteration of these biologic processes may affect BP control; slowed metabolism of endogenous hormones or co-administered medications may yield variable clinical effects between patient populations [5]. These metabolic profiles can be leveraged to identify new pharmacogenomic variants that underlie these changes [6] and may ultimately allow for prediction of drug effectiveness and safety based upon a more complete understanding of the underlying biology [7].

**Fig. 1:**
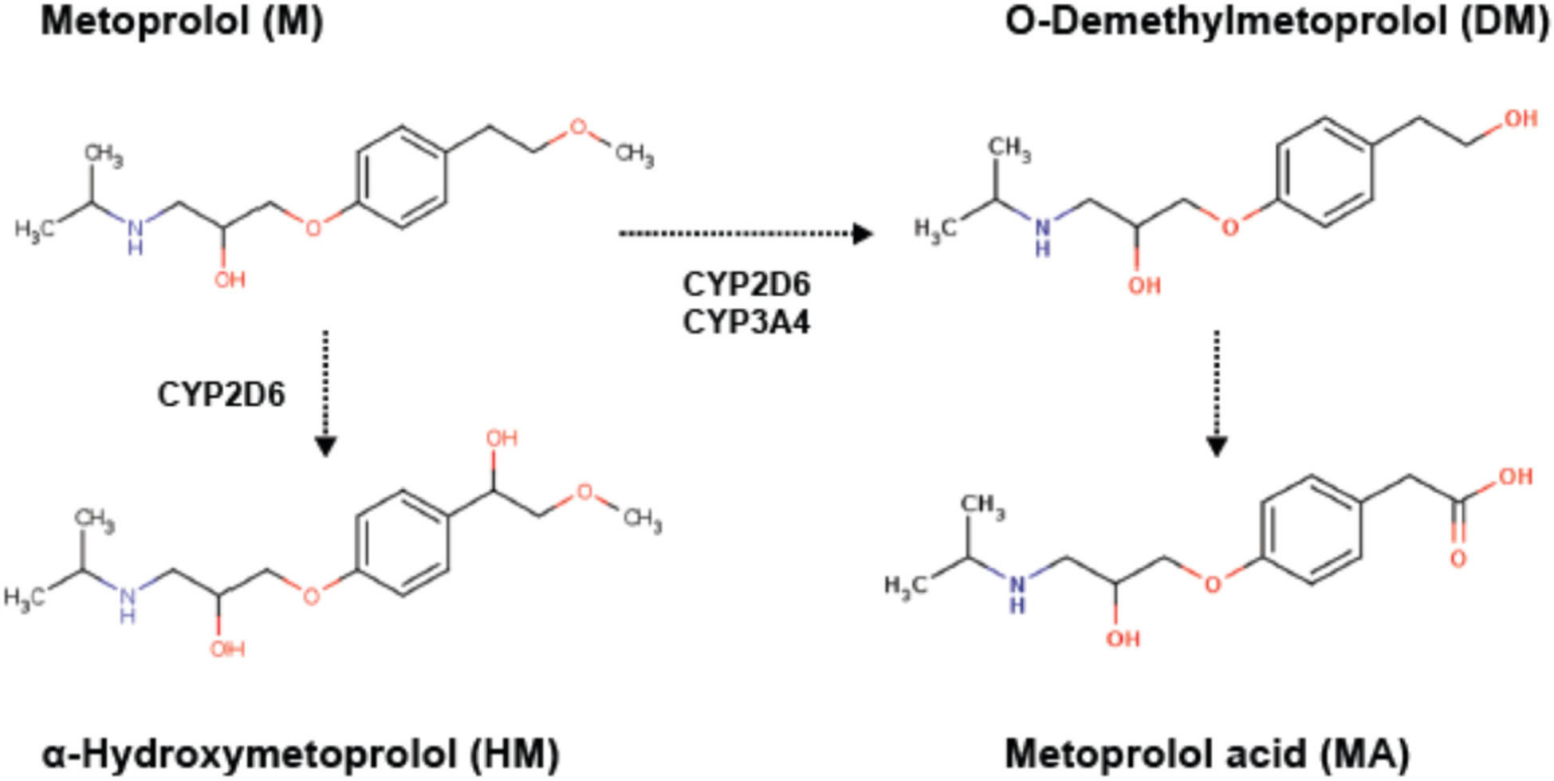
Principal pathways of metoprolol metabolism. Metoprolol is primarily metabolized to α-hydroxymetoprolol (HM) and O-demethylmetoprolol (DM) by hepatic CYP2D6 and to a lesser extent CYP3A4. O-demethylmetoprolol (DM) subsequently undergoes rapid oxidation to form metoprolol acid (MA)

Metoprolol is a beta-blocker with the same mechanism of action for BP control as atenolol. However, metoprolol is primarily metabolized through a saturable metabolic pathway, hepatic cytochrome 2D6 (CYP2D6), that is responsible for the metabolism of approximately 25% of all xenobiotics in addition to many endogenous hormones. Metoprolol is primarily metabolized to α-hydroxymetoprolol and O-demethylmetoprolol by CYP2D6 (Fig. 1). Approximately 85% of metoprolol metabolites are excreted in the urine, as well as a small amount of unmetabolized drug, making urine an ideal biofluid for monitoring. Examination of metabolites from patients treated with metoprolol may allow insight into mechanisms of this drug’s effectiveness while confirming the observations of the PEAR study that utilized a different drug with a similar mechanism. We hypothesize that variable metabolic processes between patients will be associated with metoprolol effectiveness. Thus, the primary objective of this study was to determine metabolomics markers of metoprolol effectiveness and safety in a cohort of patients initiating metoprolol therapy for BP control.

## Results

### Patient recruitment

We enrolled 86 subjects between October 1, 2013, and September 1, 2017 (Table 1). Nine subjects were lost to follow-up over the course of the study, but their samples are analyzed as part of this cohort because they had at least two urine samples with clinical outcomes available at those visits. A total of 283 urine samples were analyzed in the study.

**Table 1:** Demographics of metoprolol urine metabolomics cohort

Demographic variable	Summary statistic
Median age (IQR)	53 (46, 61)
Gender, *n* (%)	
Male	54 (62.8%)
Female	32 (37.2%)
Race, *n* (%)	
African American/Black	38 (44.2%)
American Indian/Alaskan Native	1 (1.2%)
Asian	2 (2.3%)
Caucasian/White	43 (50.0%)
Mixed race	2 (2.3%)
Hispanic/Latino	12 (14.0%)

### Drug effectiveness and safety

Overall, 58 (67.4%) patients achieved SBP control and defined as 10% decline from SBP at enrollment. Fifty-one (59.3%) achieved SBP control with metoprolol and 7 (8.1%) achieved control on additional medications following study conclusion. There were 23 adverse drug events reported during the study protocol. There were eleven visits in seven patients in which the subject had a HR less than 50 bpm; five visits in three patients in which subjects reported lightheadedness; six visits in six patients with abdominal pain, nausea, or vomiting, and one in which a subject reported sexual disturbance. None of the bradycardic patients experienced lightheadedness and all were asymptomatic. Only one episode of lightheadedness resulted in discontinuation of the study drug, lisinopril.

### Identification of metoprolol metabolites in patient samples

Multivariant data analysis subjected to unsupervised PCA-X analysis using metoprolol status as a classifier did not support group separation (Fig. 1 Suppl). However, supervised OPLS-DA showed good separation of patients on metoprolol from patients not taking the drug (Fig. 1 Suppl B). The representative loadings S-plot generated from the OPLS-DA model and revealed, as expected, that several metoprolol metabolites significantly contribute to clustering (Fig. 1 Suppl C). Seventeen major features/ions driving separation were identified on the S-plot and related information is shown in Table 2. Metoprolol, hydroxymetoprolol, and metoprolol acid ions correspond to points #2, #8, and #14, respectively.

**Table 2:** Putative urinary metabolites identified by multivariate data analysis using metoprolol status as a classifier

ID	Name	*m*/*z*	*p* (Corr) [1]	ESI	RT (min)	Adduct	Mass	Formula	Delta (ppm)
1	Pyrocatechol sulfate/catechol sulfate	189.996	− 0.309	Neg	0.386	[M − H]−	188.989	C6H6O5S	14
2	Metoprolol	267.184	− 0.306	Pos	0.443	[M + H]+	268.191	C15H25NO3	1
3	Hydroxyhippuric acid	195.054	− 0.214	Neg	0.628	[M − H] −	194.046	C9H9NO4	1
4	Hippuric acid	179.059	− 0.338	Neg	0.643	[M − H] −	178.051	C9H9NO3	0
5	Unknown	179.133	− 0.305	Neg	0.671				
6	Acetylmethylpyridine	135.069	− 0.350	Neg	0.686	[M − H] −	134.061	C8H9NO	1
7	Methoxyspirobrassinol	282.049	− 0.355	Pos	0.729	[M + H]+	283.057	C12H14N2O2S2	0
8	Hydroxymetoprolol	283.178	− 0.398	Pos	0.743	[M + H]+	284.186	C15H25NO4	1
9	Methyluric acid	182.045	− 0.320	Neg	1.944	[M − H] −	181.037	C6H6N4O3	1
10	Quinic acid	192.064	− 0.293	Neg	2.116	[M − H] −	191.056	C7H12O6	1
11	Glucose/fructose/galactose/myo-inostitol	180.064	− 0.313	Neg	2.144	[M − H] −	179.056	C6H12O6	0
12	Dimethylphenol	122.074	− 0.406	Neg	2.159	[M − H] −	121.066	C8H10O	1
13	Tigloidine/dumetorine/dihydrodioscorine	223.158	− 0.392	Neg	2.187	[M − H] −	222.150	C13H21NO2	0
14	Metoprolol acid	267.146	− 0.422	Pos	3.317	[M + H]+	268.154	C14H21NO4	1
15	Tigloidine/dumetorine/dihydrodioscorine	223.158	− 0.374	Neg	3.332	[M − H] −	222.150	C13H21NO2	0
16	Glutamine	146.069	0.350	Neg	1.065	[M − H] −	145.062	C5H10N2O3	1
17	Phenylacetylglutamine	264.111	0.321	Neg	1.065	[M − H] −	263.103	C13H16N2O4	3

Abbreviations: *m/z* mass to charge ratio, *p (Corr) p* value of the correlation, *ESI* electrospray ionization, *RT* (min) retention time in minutes, *ppm* parts per million

**Fig. 2:**
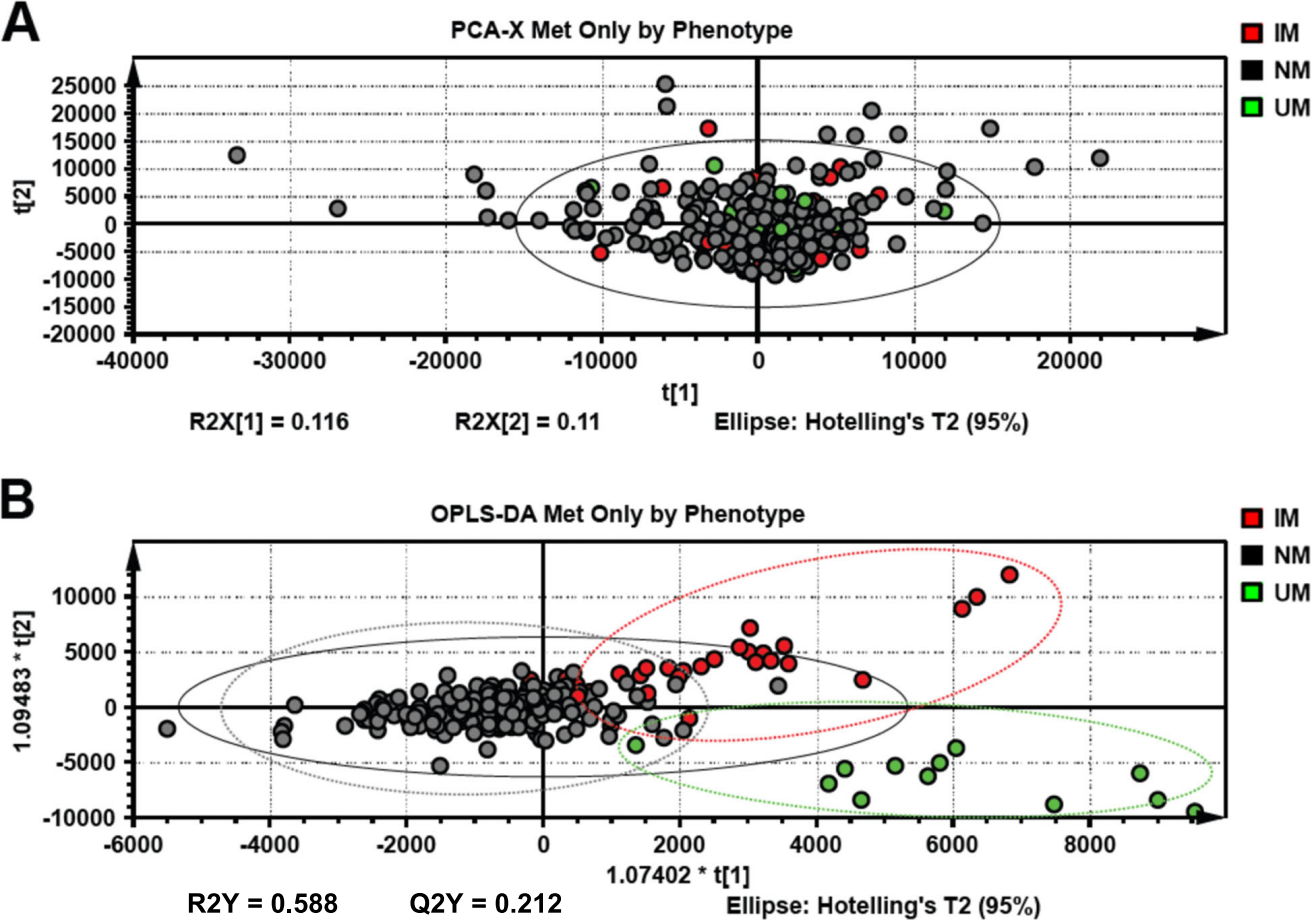
Multivariant data analysis of LC/MS-derived metabolomics data using the patient CYP2D6 phenotype. Urinary metabolomics data was subjected to unsupervised PCA-X data analysis using the patient phenotype as a classifier (**a**). Normal metabolizer (NM), intermediate metabolizer (IM), and ultra-rapid metabolizer (UM) phenotypes are denoted in black, red, and green, respectively. Scores scatter plot of supervised orthogonal projection to latent structure discriminant analysis (OPLS-DA) model using patient phenotype shows group clustering by phenotype (**b**). All data were normalized to urine creatinine abundance

All major urinary metabolites were identified including hydroxymetoprolol (*m*/*z* = 283.178, RT = 0.74), metoprolol acid (*m*/*z* = 267.146, RT = 3.32), O-demethylmetoprolol (*m*/*z* = 254.175, RT 0.74), as well as unmetabolized metoprolol (*m*/*z* = 267.184, RT = 0.44). An ion matching the expected mass of metoprolol glucuronide was also identified (*m*/*z* = 444.187, RT = 4.62). Creatinine-normalized ion abundance values and statistical analysis for all metoprolol metabolites, microbial metabolites, and other unidentified metabolites are found in Figure 2 Suppl A, and Suppl B, respectively. Patient metoprolol dose was also correlated to metabolite abundance (Fig. Suppl 6A). As expected, excreted metoprolol metabolite abundances, on average, increased with dose. There was significant variation within each dose suggesting that, despite being on an extended-release formulation, time after dosage greatly influenced urinary concentrations. Additionally, co-administration of other CYP2D6 metabolized drugs did not impact the abundance of metoprolol metabolites (Fig. Suppl 6B).

### Metabolomic profiling reveals elevated microbial-derived metabolites in response to metoprolol therapy

MVA analysis by OPLS-DA led to the identification of many features that drive separation during supervised analysis dependent on metoprolol status (Fig. 1 Suppl A & Suppl B). Features contributing greatest to group clustering were features/ions #3 and #4 as denoted in the S-plot analysis (Fig. 1 Suppl B). These ions correspond to hippuric acid and hydroxyhippuric acid, respectively, and as outlined in Table 2. Hippuric acid is a normal component of human urine and is formed by the conjugation of benzoic acid and glycine by microbial metabolism in the gut. Hydroxyhippuric acid is also considered a microbial derived end-product and both originate from polyphenol metabolism by intestinal microflora. Feature #9 is another microbial-dependent metabolite, methyluric acid (Table 2). All three compounds are significantly elevated in patients taking metoprolol. These compounds reflect gut flora composition and urinary levels are reduced in several pathological conditions, including patients with Crohn’s disease and impaired glucose tolerance [8, 9].

### Patient CYP2D6 phenotypes differentially impact urinary metoprolol metabolite concentrations

Unsupervised PCA-X analysis using metoprolol positive samples and patient phenotype as a classifier did not support group separation (Fig. 2a). Scores scatter plot based on the OPLS-DA model showed significant clustering dependent on patient phenotype (Fig. 2b). Metoprolol metabolite abundance was compared in all patient samples and analyzed by CYP2D6 metabolizer phenotype, namely IM, NM, and UM (Fig. 3 Suppl A) and CYP2D6 activity scores (Fig. 4 Suppl B). Unmetabolized metoprolol and metoprolol glucuronide concentrations decreased with increasing CYP2D6 activity. Conversely, urinary hydroxymetoprolol abundance increased with increasing CYP2D6 metabolic capacity. Comparing classification by CYP2D6 phenotype and activity score found that urinary metoprolol metabolite profiles of patients with an activity score of 2.0 more closely correlate to UM patients than other NM activity score groups, namely 1.0 and 1.5 (Fig. 4 Suppl B).

### Metoprolol metabolite ratios strongly correlate to CYP2D6 phenotype and activity scores

Metabolite concentrations varied greatly within each group and appear to be influenced significantly by elapsed time between collecting urine samples and when a patient took their dose of metoprolol. To assess whether genotype influences metabolite abundance within given samples, metoprolol metabolite ratios were determined by comparing normalized abundance values for each metabolite within the same patient sample at a given visit. Although urinary concentrations for any given metabolite varied between patient and visit samples, the relative amount of each metabolite was consistent with metabolite ratios. Patient metoprolol metabolites were normalized to unmetabolized metoprolol (M) (Fig. 5 Suppl A). Metoprolol to demethylmetoprolol (DM) ratios (M to DM) and metoprolol to hydroxymetoprolol (HM) ratios (M to HM) closely correlated to increasing CYP2D6 activity. An inverse relationship was observed when samples were normalized to metoprolol acid (MA) and ratios decreased with reduced CYP2D6 capacity as reflected by patient phenotype and activity scores (Fig. 4 Suppl B). The fact that metoprolol metabolites can quickly be used to provide insight into CYP2D6 genotype independent of dose or time after the dosage is interesting. These ratios are also independent of age, sex, diet, and other confounding factors reflected in the observed metabolomic heterogeneity between patient samples.

### Metabolomic analysis did not identify metabolite alterations dependent on blood pressure or heart rate response

Identification of metabolic biomarkers associated with either patients reaching BP or HR response goals as defined in the “Materials and methods” section was assessed using MVA analyses. PCA model scores scatter plots did not reveal pronounced separation by either BP or HR as a classifier (data not shown). Supervised OPLS-DA models also did not support clustering based on either classifier (Fig. 5 Suppl A & Suppl B). Although not statistically significant, hippuric acid levels, a gut microbiota-derived metabolite, trended towards being elevated in patients who reached BP goals at the time of visit (*p* = 0.061) (data not shown).

### Limitations

The numbers of the more uncommon *CYP2D6* genotypes, PM and UM, are low in this cohort, which limits our ability to identify endogenous metabolites associated with *CYP2D6* genotype. While this study did not identify metabolites associated with metoprolol effectiveness, this may be due to examination in only 287 samples. There may indeed be metabolites associated with drug effectiveness if more patients and samples are examined. However, we were powered to detect metabolites with more than 95% power when advised by drug ingestion and *CYP2D6* genotype. The risk of type one errors associated with multiple comparisons remains, though we have used methods to account for these comparisons and repeated experiments to confirm results. This pragmatic trial did not control patient diets, and thus, subtle changes in endogenous metabolites may be masked by underlying dietary changes. However, our findings support prior investigators work at other facilities in which, presumably, diets would be equally variable.

In addition to the impact of diet on metabolite profiles, differences in metabolism between individuals may increase metabolite variability resulting in poor model prediction. Urine samples analyzed in this study come from a somewhat heterogeneous patient population, which varied by age, sex, and race, all of which can significantly impact metabolism between individuals. Metabolic differences between patients, as well as patient’s diets prior to sample collection, could increase metabolite variability and negatively impact statistical analyses and modeling. Despite there being a large amount of variation between urinary metabolite profiles of patient samples collected in the study, several ions/features were identified.

We have considered the complex phenotypes of blood pressure and heart our dominant phenotypes to be examined, though we did not perform phenomics to confirm that these phenotypes drive our metabolomic separation. Ultimately, this study was designed to examine SBP and HR, and thus, while other phenotypes, such as diabetes, may influence the results, these were the main outcomes of our study and thus are the primary phenotype examined. Additionally, changes in microbiome and lipid metabolism due to beta-blocker therapy are mechanisms supported by known drug actions.

## Discussion

Metabolomic analyses of serial samples of patients on metoprolol treatment for uncontrolled HTN were not able to identify unique metabolites associated with drug effectiveness. Metoprolol metabolites segregated very well with CYP2D6 phenotype, suggesting that drug metabolism can be predicted based upon knowledge of *CYP2D6* genotype and the presence of drug-drug interactions. However, these data are still limited by small numbers of PM and UM genotypes in the study. This may have contributed to our inability to identify endogenous metabolites predictive of clinical effectiveness, if CYP2D6 drug metabolism plays a major role in drug effectiveness, as hypothesized. The metabolites are a result of both genetic and environmental factors in this pragmatic trial. We believe this because some of the identified metabolites are diet-derived while some clearly segregate to the metabolic pathways examined. We have taken care to repeat experiments and used methods to account for the multiple comparisons present in these analyses, though the risk of type one errors remains given the number of metabolites examined. Additional analysis examining the composite outcome of 10% SBP or HR decline may yield association, as it did in the phenotyping analysis [10]. We will increase the numbers of lower frequency genotypes in this cohort and power for this outcome in the future.

Analysis of metabolomics data identified elevated levels of hippuric, hydroxyhippuric, and methyluric acids in patients taking metoprolol. These compounds are all considered gut flora-derived metabolites suggesting that prolonged metoprolol treatment may influence microbial composition and diversity within the GI. There is increasing evidence that gut flora dysbiosis may contribute to hypertension [11]. Moreover, metagenomic analysis of fecal samples from patients with atherosclerotic cardiovascular disease found metoprolol therapy positively correlated with alterations in metagenomic linkage groups (MLGs) [12] supporting the drug may affect the microbiome. A recent study suggested that hippuric acid is a metabolomic marker for gut microbiome diversity and found decreased hippuric acid is associated with metabolic syndrome [13]. Studies have also found that reduced urinary hippuric acid levels in Crohn’s disease patients are diet-independent and instead dependent on intestinal microbial metabolism [8]. Another study found that patients with impaired glucose tolerance exhibited decreased excretion of methyluric acid in addition to hippuric acid and hydroxyhippuric acid [13]. Whether these gut flora-associated metabolites are indicators of microflora health or actively influence health is yet to be determined. Metoprolol therapy elevates urinary excretion of all three of these gut flora-associated metabolites suggesting in the drug either directly or indirectly influences gut flora composition.

Our findings expand upon the findings of the PEAR cohort. We were unable to confirm their findings of alteration in β-alanine pathway metabolites amongst African American subjects. Aside from the diagnosis of diabetes, we did not have detailed glucose data in these subjects to further stratify the racial groups by the presence of hyperglycemia. Urine is not the ideal biologic matrix to examine changes in glucose since urinary excretion is dependent upon the relatively high tubular reabsorption threshold of glucose and the presence/absence of renal insufficiency. Thus, our data may support their finding if further stratified in this way and blood glucose is examined in our additional samples.

In summary, in the treatment of HTN, metoprolol therapy appears to alter the gut microbiome and the composition of the microbiome may be an important factor in the effectiveness of antihypertensive drugs. Further work should focus on the stratification of drug effectiveness and gut microbiome composition in order to understand the biologic interactions between these systems.

## Materials and methods

### Study design and setting

This was a prospective pragmatic trial (NCT02293096) that enrolled patients with uncontrolled HTN from local clinics, the University of Colorado Emergency Department (ED), and the local community. The study was approved by the Colorado Multiple Institution Review Board.

### Subjects

We enrolled subjects with uncontrolled HTN between 30 and 80 years of age. Exclusion criteria included end-stage liver disease, glomerular filtration rate < 60 ml/min/1.73 m^2^, pregnancy, American Association of Anesthesiologists (ASA) classification of > 3, prisoners or wards of the state, decisionally challenged, heart rate (HR) < 60 beats per minute, AV block > 240 msec, active reactive airway disease, illicit drug use in the preceding 30 days (excluding marijuana), allergy to metoprolol succinate, or severe peripheral arterial circulatory disorders. If the subject was consented in the ED, the acute medical condition was treated and stabilized, and they followed up in the study clinic 1 week after the ED visit. Subjects were followed for up to 6 weeks; medication reconciliation, HR, BP, and urine samples were collected at these weekly visits.

### Drug intervention

Subjects without allergy or intolerance were started on the angiotensin-converting enzyme (ACE) inhibitor lisinopril, 10 mg daily, as first-line therapy if they were not already taking an ACE inhibitor or an angiotensin receptor blocker class. If BP remained uncontrolled, defined as > 140/90 mmHG, after taking at least five doses of the therapy, then metoprolol succinate was added. Subjects were followed weekly and metoprolol was up-titrated for 4 weeks, as tolerated by BP and HR. No further up-titration was allowed if the BP was < 140/90 mmHG or the HR was less than 50 beats per minute. The primary outcome was a systolic blood pressure (SBP) decline of 10% or more, from baseline SBP, at 4 weeks following metoprolol succinate therapy. Secondary outcomes included a composite of HR or BP control, defined as a 10% decline in SBP or HR at 4 weeks of therapy, and the presence of adverse drug events (ADEs) associated with metoprolol succinate therapy. Adverse drug events included abdominal pain, nausea/vomiting, HR less than 50 beats per minute, reactive airway disease exacerbation, dizziness/lightheadedness, sexual dysfunction, myocardial infarction, or congestive heart failure. Subjects continued their other medications and could eat their typical diet during the protocol.

### CYP2D6 genotyping

Genomic DNA was extracted from whole blood via the Puregene® Blood Core Kit B (Qiagen) according to the manufacturer’s instructions. *CYP2D6* was genotyped using the Multiplex SNaPshot technique previously described [14]. This assay detects 20 *CYP2D6* clinically significant variants and identifies copy number variants. While other hepatic cytochromes contribute to metoprolol metabolism, namely CYP3A4, CYP2B6, and CYP2C9 [15], these isoforms contribute to less than 20% of the drug’s metabolism; thus, we have focused on CYP2D6 since it is the primary pathway of metabolism. Genotyping was performed after subjects completed the protocol; thus, the investigators were blinded to the *CYP2D6* genotype during treatment. Predicted phenotypes were determined utilizing CYP2D6 activity score, as described by Gaedigk et al. [16]. Each identified CYP2D6 SNV was assigned a predicted enzyme activity score [16, 17]. Gene deletions were designated as an activity score of zero. The predicted enzyme phenotype was determined by addition of the individual gene activity scores, accounting for gene copies yielding decreased enzyme activity and gene duplications in each patient. A score of 0 was predicted to be a poor metabolizer (PM), 0.5 was predicted to be intermediate metabolizer (IM), 1.0–2.0 was predicted to be a normal metabolizer (NM), and 2.5 or greater was predicted to have an ultra-rapid metabolizer (UM) phenotype. Genotypes were confirmed with known reference genotype samples from 5 PMs, 4 IMs, and 24 NMs [18, 19]. Copy number variations were determined by TaqMan Copy Number Assay (Life Technologies, CA) and then by pyrosequencing allele quantification in the known samples [18].

### Metabolomic analyses

Metoprolol and related metabolites are primarily excreted in the urine. The identification of these compounds in urine by LC/MS has been described extensively [20–22]*.* Urinary metabolite abundance was determined using predicted *m*/*z* values and associated peak intensities. Prospective metabolites were initially identified by *m*/*z* values, then verifying an absence of the corresponding ion in “untreated” samples. Characteristic fragmentation peaks were identified by expanding target ion peaks using MassLynx software. Potential fragment peaks were compared with those confirmed experimentally by targeted LC-MS/MS peaks found on the METLIN database when possible. Urine from each study visit was aliquoted and frozen at − 80 °C within an hour of the study visit. Samples were shipped on dry ice to the NIH Laboratory of Metabolism at the National Cancer Institute. Samples were thawed on ice and deproteinated (dilution of 1:6) using a solution of isopropanol/acetonitrile/water (65/30/5) containing α-aminopimelic acid as an internal standard for hydrophilic interaction liquid chromatography (HILIC). All samples were vortexed for 30 s and spun at 15 k × *g* for 15 min to remove the precipitant. Cleared supernatants were transferred into a 96-well plate for metabolite extraction. A Microlab Starlet automated liquid handler (Hamilton Robotics) was used for subsequent pipetting and dilutions. For HILIC analysis, samples were randomized and an aliquot (5 μL) was injected into a 2.1 × 50 mm Acquity UPLC BEH amide column (1.7 μm) attached to a Waters Acquity H-class UPLC system for chromatographic separation. The UPLC system consisted of a quaternary solvent manager, FTN-solvent manager, and a column manger, all controlled by MassLynx Software (Waters Corporation). Metabolite separation was achieved using a mobile phase mixture of 10 mmol/L ammonium acetate in 90% acetonitrile (A, pH = 9.0) and 10 mmol/L ammonium acetate in 10% acetonitrile (B, pH = 9.0). A gradient elution was performed over 10 min using 1 to 60% B in 4 min, 60 to 80% B at 8 min, holding at 80% B to 8.5 min, returning to initial conditions for column equilibration. The flow rate was maintained at 0.4 mL/minute, and the total run time for each sample was 12.5 min. The column temperature was maintained at 40 °C. Mass spectrometric analysis was performed on a Waters XEVO G2 ESI-QTOF mass spectrometer (Waters Corporation) in both positive and negative ionization modes. Sulfadimethoxine was used as the lock mass (*m*/*z* 311.0814+) for accurate mass calibration in real time. Pooled samples and standard mix were also injected recurrently during the run as quality control to monitor the stability of the system. MassLynx software (Waters Corporation) was used to acquire mass chromatograms and mass spectral data in centroid format.

### Data processing, multivariate data analysis, and metabolite identification

Retention time alignment and peak picking were performed on chromatographic and spectral data using Progenesis QI software (Nonlinear Dynamics, Newcastle, UK). Sample data matrixes were generated and normalized to urine creatinine ion abundance. Features/ions identified in positive and negative ionization modes were combined into a single file using a custom R script (Supplement B). Alterations in systemic urinary metabolites were compared by multivariate data analysis (MVA) using SIMCA software (Version 14) (Umetrics, Kinnelon, NJ, USA). Normalized data was subjected to unsupervised principal component analysis (PCA-X) to visualize sample and group clustering. Specific features/ions that contribute to group clustering were identified using supervised orthogonal projection to latent structures-discriminant analysis (OPLS-DA) and S-plot analysis. To assess changes in endogenous metabolites, metoprolol and other drug metabolites were removed from prior to SIMCA analysis. Significant features identified by OPLS-DA were identified by database searches. These searches were performed using METLIN [23] and Human Metabolome Database (HMDB) databases [24] by *m*/*z*, retention times, and fragmentation patterns, when available.

### Statistical analysis

Statistical analysis was performed using GraphPad Prism software (Version 7.03) (GraphPad Software, San Diego, CA, USA). Outliers were identified, and normality assessed using ROUT and D’Agostino-Pearson tests, respectively. Non-parametric (Mann-Whitney) or one-way ANOVA (Kruskal-Wallis) tests were performed on data sets to determine statistical significance. Dunn’s multiple comparison test was used for post hoc analyses. Differences were considered significant for adjusted *p* values less than 0.05. Supervised OPLS-DA was used to assess whether the patient phenotype was sufficient to drive metabolite group separation. Raw data were generated at the University of Colorado School of Medicine and the National Institutes of Health. Derived data supporting the findings of this study are available from the corresponding author (AAM) upon request.

## Supplementary information

**Additional file 1: Figure 1 Suppl.** Impact of metoprolol therapy on global metabolite profiles. **Figure Suppl.** Abundance of metoprolol- and microbiota-dependent metabolites in patient urine. **Figure 3 Suppl.** CYP2D6 phenotype differentially impacts metoprolol metabolite concentrations in urine. **Figure 4 Suppl.** Urinary metoprolol metabolite ratios reflect CYP2D6 genotype. **Figure 5 Suppl.** Effect of systolic blood pressure or heart rate response on metabolomic profiles. **Figure 6 Suppl.** Impact of metoprolol dose and CYP2D6 drug co-medication on metoprolol metabolite abundance.

**Additional file 2.** Supplement B: Features/ions identified in positive and negative ionization modes were combined into a single file using a custom R script.
