# Supplementary material for: Metabolomic profiling of metoprolol hypertension treatment reveals altered gut microbiota-derived urinary metabolites
Source: Hum Genomics. 2020 Mar 11;14:10. doi: 10.1186/s40246-020-00260-w (PMC7066769; doi:10.1186/s40246-020-00260-w)
Supplement: Supplementary file 1 — Additional file 1: Figure 1 Suppl. Impact of metoprolol therapy on global metabolite profiles. Figure Suppl. Abundance of metoprolol- and microbiota-dependent metabolites in patient urine. Figure 3 Suppl. CYP2D6 phenotype differentially impacts metoprolol metabolite concentrations in urine. Figure 4 Suppl. Urinary metoprolol metabolite ratios reflect CYP2D6 genotype. Figure 5 Suppl. Effect of systolic blood pressure or heart rate response on metabolomic profiles. Figure 6 Suppl. Impact of metoprolol dose and CYP2D6 drug co-medication on metoprolol metabolite abundance. [file 40246_2020_260_MOESM1_ESM.docx]

**R2Y = 0.257 Q2Y = -0.00367**

# Figure 1 Suppl. Impact of metoprolol therapy on global metabolite profiles.

Multivariant data analysis subjected to unsupervised PCA-X analysis using metoprolol status as a classifier (**A**). Scores scatter plot (**B**) and S-plot (**C**) of supervised orthogonal projection to latent structures-discriminant analysis (OPLS-DA) model. Features/ions of interest, including Metoprolol metabolites, are highlighted in red and correspond data outlined in **Table 2**. Features on S-plot are colored according to ion retention time (rt) (red = higher rt; blue = lower rt). All data were normalized to urine creatinine abundance.


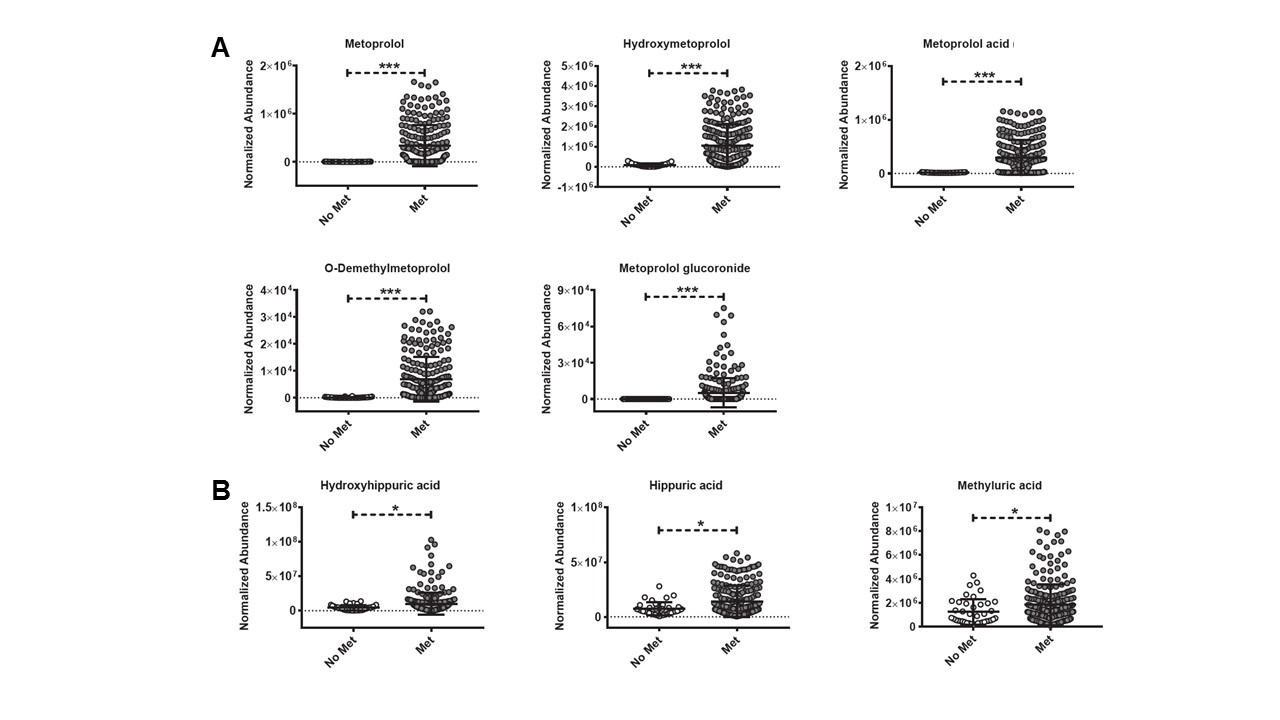


# Figure 2 Suppl. Abundance of metoprolol- and microbiota-dependent metabolites in patient urine.

The relative abundance of metoprolol metabolites, namely metoprolol, hydroxymetoprolol, metoprolol acid, O-demethylmetoprolol, and metoprolol glucuronide were determined in urine samples from patients receiving (Met) and not receiving (No Met) metoprolol therapy (**A**). Microbiota-dependent metabolites including hydroxyhippuric, hippuric, and methyluric acids were also measured and found to be significantly elevated in patients on metoprolol therapy (**B**). Differences were considered significant if p < 0.05 using non-parametric, Mann-Whitney tests. All data were normalized to urine creatinine abundance.


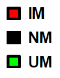


**A**

**B**

# Figure 3 Suppl. CYP2D6 phenotype differentially impacts metoprolol metabolite concentrations in urine.

Metoprolol metabolites, namely metoprolol (M), O-demethylmetoprolol (DM), hydroxymetoprolol (HA), metoprolol acid (MA), and metoprolol glucuronide (MG) were identified for each sample and analyzed by patient phenotype (**A**). CYP2D6 activity scores were also analyzed by metoprolol metabolite abundance (**B**). Normal metabolizer (NM), intermediate metabolizer (IM), and ultra-rapid metabolizer (UM) phenotypes are denoted in black, red, and green, respectively. Differences were considered significant if p < 0.05 using non-parametric, Mann-Whitney tests. All data were normalized to urine creatinine abundance.

**B**

**A**

# Figure 4 Suppl. Urinary metoprolol metabolite ratios reflect CYP2D6 genotype.

To determine whether patient genotype influences relative ratios of urinary metoprolol metabolites, metabolites within each sample were normalized to the corresponding metoprolol (M) or metoprolol acid (MA) abundance. Metabolite ratios were then analyzed by patient phenotype (**A**) or patient activity score (**B**). Normal metabolizer (NM), intermediate metabolizer (IM), and ultra-rapid metabolizer (UM) phenotypes are denoted in black, red, and green, respectively. Differences were considered significant if p < 0.05 using non-parametric, Mann-Whitney tests. All data were normalized to urine creatinine abundance.


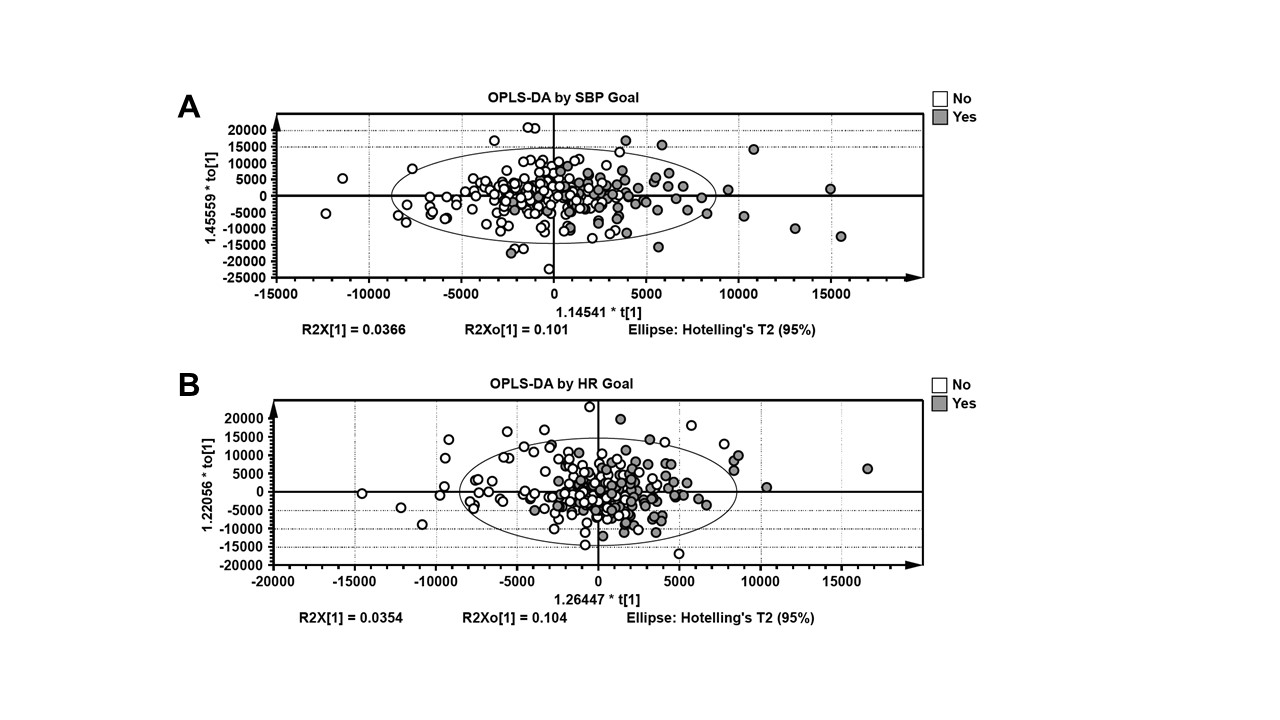


**R2Y = 0.190 Q2Y = -0.0951**

**R2Y = 0.271 Q2Y = -0.0623**

# Figure 5 Suppl. Effect of systolic blood pressure or heart rate response on metabolomic profiles.

Scores scatter plots from supervised orthogonal projection to latent structures-discriminant analyses (OPLS-DA) models based on systolic blood pressure (SBP) (**A**) or heart rate (HR) (**B**). Classification was based on whether a patient reached SBP or HR response goals at the time of visit. No statistically significant differences in urinary metabolites correlated to either SBP or HR response. Differences would have been considered significant if p < 0.05 using non-parametric, Mann-Whitney tests. All data were normalized to urine creatinine abundance.


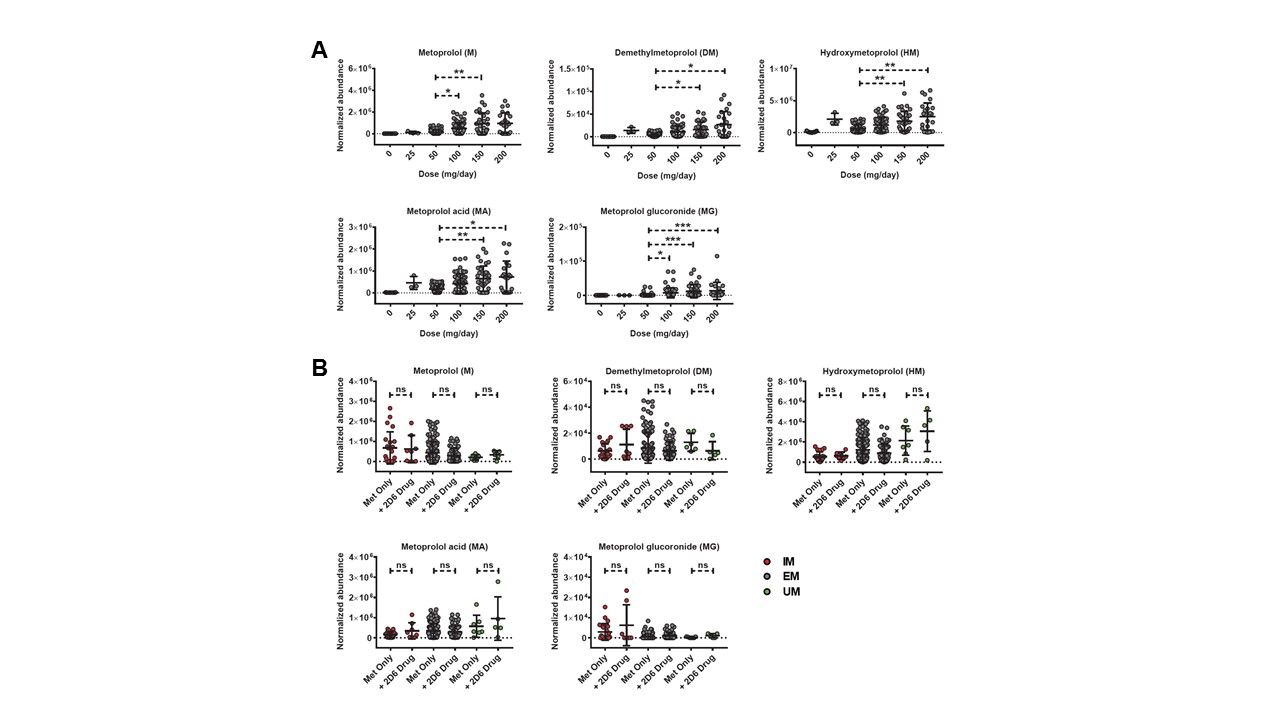

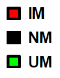


# Figure 6 Suppl. Impact of metoprolol dose and CYP2D6 drug co-medication on metoprolol metabolite abundance.

Patients in the cohort were taking 25, 50, 100, 150, or 200 mg of metoprolol per day. Metoprolol metabolite abundance positively correlated with increased metoprolol dosage (**A**). Metoprolol metabolite abundance was also measured in patients to assess whether co-treatment with additional CYP2D6 metabolized drugs influences metoprolol metabolism, (**B**). Differences would have been considered significant if p less than 0.05 using non-parametric, Mann-Whitney tests. All data was normalized to urine creatinine abundance.
