# Supplementary material for: Metabolomic profiling of metoprolol hypertension treatment reveals altered gut microbiota-derived urinary metabolites
Source: Hum Genomics. 2020 Mar 11;14:10. doi: 10.1186/s40246-020-00260-w (PMC7066769; doi:10.1186/s40246-020-00260-w)
Supplement: Supplementary file 2 — Additional file 2. Supplement B: Features/ions identified in positive and negative ionization modes were combined into a single file using a custom R script. [file 40246_2020_260_MOESM2_ESM.docx]

**Supplement B:**

**Features/ions identified in positive and negative ionization modes were combined into a single file using a custom R script.**

##Add Pos mz to Neg annotated diffreport and Neg mz to Pos annotated diffreport

setwd("M:/Home/Combine/Test")

###Step:1 Annotate Ions that appear in both Pos and Neg Mode #######

"1.69_509.3475n"

NegDataRaw<-read.csv("RPLC_Neg_Raw.csv", row.names = 1)

PosDataRaw<-read.csv("RPLC_Pos_Raw.csv", row.names = 1)

TEST1<-ProgenCombineNegPos(NegDataRaw = read.csv("RPLC_Neg_Raw.csv", row.names = 1),

PosDataRaw = read.csv("RPLC_Pos_Raw.csv", row.names = 1),

ISTDNorm = FALSE,

NegISTD ="6.26_275.0255m/z",

PosISTD = "6.26_276.0339n",

ppmtol = 5,

rttol = 2,

QCFilter = FALSE,

QCTol = 0.3,

filename = " RPLC_Combined")

ProgenCombineNegPos<-function(NegDataRaw, PosDataRaw, ISTDNorm, NegISTD, PosISTD, ppmtol, rttol, QCFilter, QCTol, filename)

{

if (missing(NegDataRaw))

stop("Need to add NegDataRaw.")

if (missing(PosDataRaw))

stop("Need to add PosDataRaw.")

if (missing(NegISTD))

stop("Need to add NegISTD.")

if (missing(PosISTD))

stop("Need to add PosISTD.")

if (missing(ppmtol))

stop("Need to add ppmtol value.")

if (missing(rttol))

stop("Need to add rttol value.")

if (missing(QCFilter))

stop("Need to add TRUE/FALSE to QCFilter")

if (QCFilter)

if (exists(colnames(PosDataRaw[,grep("*Pool*",colnames(PosDataRaw))])))

if (exists(colnames(NegDataRaw[,grep("*Pool*",colnames(NegDataRaw))])))

stop("Need to add 'Pool' to Sample Names of Pooled Samples")

if (QCFilter)

if(missing(QCTol))

stop("Need to add QCTol.")

if (ISTDNorm)

if(missing(NegISTD))

stop("Need to add NegISTD.")

if (ISTDNorm)

if(missing(PosISTD))

stop("Need to add PosISTD.")

dir.create("Troubleshooting", showWarnings = FALSE)

##Change Column Names##

RawColNames<-colnames(PosDataRaw[,1:3])

colnames(PosDataRaw)[1:3]<-c("m","mz","rt")

colnames(NegDataRaw)[1:3]<-c("m","mz","rt")

##Obtain Run "Code"##

RunCodeNeg<-strsplit(as.character(colnames(NegDataRaw)),"_")##string split

RunCodeNeg[1:3]<-NULL##remove first 3

RunCodeNeg<-as.factor(paste(sapply(RunCodeNeg, "[[",1), sep = "_")) ##obtain group labels

RunCodeNeg<-levels(RunCodeNeg)

RunCodeNeg<-paste0("*",RunCodeNeg,"*")

RunCodePos<-strsplit(as.character(colnames(PosDataRaw)),"_")##string split

RunCodePos[1:3]<-NULL##remove first 3

RunCodePos<-as.factor(paste(sapply(RunCodePos, "[[",1), sep = "_")) ##obtain group labels

RunCodePos<-levels(RunCodePos)

RunCodePos<-paste0("*",RunCodePos,"*")

NegMZ<-NegDataRaw[grep("*m/z",rownames(NegDataRaw)),]

NegMZ$m<-NegMZ$mz+1.0078

NegDataRaw<-NegDataRaw[grep("*n",rownames(NegDataRaw)),]

NegDataRaw<-rbind(NegDataRaw,NegMZ)

PosMZ<-PosDataRaw[grep("*m/z",rownames(PosDataRaw)),]

PosMZ$m<-PosMZ$mz-1.0078

PosDataRaw<-PosDataRaw[grep("*n",rownames(PosDataRaw)),]

PosDataRaw<-rbind(PosDataRaw,PosMZ)

######PosMode#####

##From NEUTRAL###

NegUpPPM<-sapply(NegDataRaw$m,function(x) x*ppmtol/1e6+x)

NegDownPPM<-sapply(NegDataRaw$m,function(x) x*-ppmtol/1e6+x)

NegRange<-data.frame(startmzNeg = NegDownPPM,endmzNeg=NegUpPPM,startRtNeg = NegDataRaw$rt-(rttol/60)/2, endRtNeg = NegDataRaw$rt+(rttol/60)/2)

NegDataForFilter<-cbind(NegRange,NegDataRaw)

PosToFilterMZRT<-data.frame(ToFilterMZ = PosDataRaw$m,ToFilterRT = PosDataRaw$rt,PosDataRaw)

qrows<-dim(NegDataForFilter)[1]

pb <- txtProgressBar(1, qrows, style=3)

PosToFilterMZRTFinal<-c()

for (q in 1:qrows) {

subdata1<-subset(PosToFilterMZRT, NegRange[q,1] < PosDataRaw$m & PosDataRaw$m < NegRange[q,2] & NegRange[q,3] <PosDataRaw$rt & PosDataRaw$rt<NegRange[q,4])

PosToFilterMZRTFinal<-rbind(PosToFilterMZRTFinal,subdata1)

setTxtProgressBar(pb, q)

}

PosToFilterFINAL<-data.frame(PosToFilterMZRTFinal,NegMode="Found M-H")

Pos1<-data.frame(NegMode = PosToFilterFINAL[,ncol(PosToFilterFINAL)]); rownames(Pos1)<-rownames(PosToFilterFINAL)

PosDataFinal<-merge(PosDataRaw,Pos1, by = "row.names", all.x = TRUE);rownames(PosDataFinal)<-PosDataFinal[,1];PosDataFinal[,1]<-NULL

######NegMode#####

##From NEUTRAL###

PosUpPPM<-sapply(PosDataRaw$m,function(x) x*ppmtol/1e6+x)

PosDownPPM<-sapply(PosDataRaw$m,function(x) x*-ppmtol/1e6+x)

PosRange<-data.frame(startmzPos = PosDownPPM,endmzPos=PosUpPPM,startRtPos = PosDataRaw$rt-(rttol/60)/2, endRtPos = PosDataRaw$rt+(rttol/60)/2)

PosDataForFilter<-cbind(PosRange,PosDataRaw)

NegToFilterMZRT<-data.frame(ToFilterMZ = NegDataRaw$m,ToFilterRT = NegDataRaw$rt,NegDataRaw)

qrows<-dim(PosDataForFilter)[1]

pb <- txtProgressBar(1, qrows, style=3)

NegToFilterMZRTFinal<-c()

for (q in 1:qrows) {

subdata1<-subset(NegToFilterMZRT, PosRange[q,1] < NegDataRaw$m & NegDataRaw$m < PosRange[q,2] & PosRange[q,3] <NegDataRaw$rt & NegDataRaw$rt<PosRange[q,4])

NegToFilterMZRTFinal<-rbind(NegToFilterMZRTFinal,subdata1)

setTxtProgressBar(pb, q)

}

NegToFilterFINAL<-data.frame(NegToFilterMZRTFinal,PosMode="Found M+H")

Neg1<-data.frame(PosMode = NegToFilterFINAL[,ncol(NegToFilterFINAL)]); rownames(Neg1)<-rownames(NegToFilterFINAL)

NegDataFinal<-merge(NegDataRaw,Neg1, by = "row.names", all.x = TRUE);rownames(NegDataFinal)<-NegDataFinal[,1];NegDataFinal[,1]<-NULL

write.csv(NegDataFinal,paste0("Troubleshooting/",filename,"_Neg_addPos.csv"))

write.csv(PosDataFinal,paste0("Troubleshooting/",filename,"_Pos_addNeg.csv"))

#####Step 2: Combine the Pos and Neg Mode############

Neg<-data.frame(rownames(NegDataFinal),NegDataFinal)

Pos<-data.frame(rownames(PosDataFinal),PosDataFinal)

colnames(Neg)[1]<-"Compound"

colnames(Pos)[1]<-"Compound"

NegIonBoth<-Neg[grep("*Found*", Neg$PosMode),] ##subset by ions found in both Neg/Pos

PosIonBoth<-Pos[grep("*Found*", Pos$NegMode),] ##subset by ions found in both Neg/Pos

if(dim(NegIonBoth)[1]==dim(PosIonBoth)[1])

{

NegPosCombine<-data.frame(NegIonBoth$Compound, PosIonBoth$Compound) ##combine mz found in both

rownames(NegPosCombine)<-NegPosCombine$NegIonBoth.Compound ##set row names to first column

rownames(Neg)<-Neg[,1] ##set row names to mz column so same as row names of "NegPosCombine"

NegCombine<-merge(Neg, NegPosCombine, by="row.names", all.x = TRUE) ##merge Neg with NegPosCombine keeping all Neg rows

NegCombine$NegIonBoth.Compound<-sapply(NegCombine$NegIonBoth.Compound, as.matrix)

NegCombine$PosIonBoth.Compound<-sapply(NegCombine$PosIonBoth.Compound, as.matrix)

NegCombine$PosMode<-sapply(NegCombine$PosMode, as.matrix)

NegCombine[is.na(NegCombine)] <- "" ##remove NAs from NegCombine

NegCombine$Row.names<-NULL ##delete Row.names column

NegCombine<-NegCombine[order(NegCombine$Compound),] ##put Neg back in original order

rownames(NegCombine)<-NegCombine[,1]; NegCombine<-NegCombine[,-1] ##put row names back in Neg

rownames(NegPosCombine)<-NegPosCombine$PosIonBoth.Compound ##repeat for Pos mode

rownames(Pos)<-Pos[,1]

PosCombine<-merge(Pos, NegPosCombine, by="row.names", all.x = TRUE)

PosCombine$NegIonBoth.Compound<-sapply(PosCombine$NegIonBoth.Compound, as.matrix)

PosCombine$PosIonBoth.Compound<-sapply(PosCombine$PosIonBoth.Compound, as.matrix)

PosCombine$NegMode<-sapply(PosCombine$NegMode, as.matrix)

PosCombine[is.na(PosCombine)] <- ""

PosCombine$Row.names<-NULL

PosCombine<-PosCombine[order(PosCombine$Compound),]

rownames(PosCombine)<-PosCombine[,1]; PosCombine<-PosCombine[,-1]

####NegData###

##Create a subset object of integrated peaks and "mzrt" column using date as a column name pattern#

NegRaw<-Neg[,grep(RunCodeNeg,colnames(Neg))]

##Create a subset of annotations before peaks and annotations after peaks#

NegCombineAnnot<-NegCombine[,-grep(RunCodeNeg,colnames(NegCombine))] ##subset without integrated peaks

NegCombineAnnot1<-NegCombineAnnot[,1:(ncol(NegCombineAnnot)-3)]##subset before peaks

NegCombineAnnot2<-NegCombineAnnot[,(ncol(NegCombineAnnot)-2):ncol(NegCombineAnnot)]##subset after peaks

if(ISTDNorm){

##Find ISTD row##

rownames(NegRaw)

##and rename to "ISTD"##

ISTDrow<-grep(NegISTD, rownames(NegRaw))

rownames(NegRaw)[ISTDrow]<-"ISTD"

##Check rownames to confirm

rownames(NegRaw)

##convert to data matrix##

df1 = as.matrix(NegRaw)

##Divide all rows by ISTD Row (Must find ISTD Row Number)##

ISTDMean<-mean(df1["ISTD",], dims = 1)

df1["ISTD",]<-sapply(df1["ISTD",],function(x) x/ISTDMean)

ISTD<-t(t(df1)/df1["ISTD",])

NegFileName<-paste0(filename,"_Neg")

##export ISTD normalized data for records

write.csv(ISTD,paste0("Troubleshooting/",NegFileName,"_ISTD.csv"))

}else{

NegFileName<-paste0(filename,"_Neg")

ISTD<-NegRaw

}

##Determine max peak intensity in each row##

maxint<-apply(ISTD, 1, max)

NegCombineAnnot2<-data.frame(maxint,NegCombineAnnot2)

if(QCFilter){

####Step 3:Evaluate QC Variability####

QC<-t(ISTD) ##tranpose ISTD dataframe

QCtb <- QC[grep("*Pool*",rownames(QC)),] ##subset only Pool injections

QCtb<-t(QCtb) ##transpose again

QCsd<-data.frame(apply(QCtb, 1, sd)); names(QCsd)[1] <- "SD" ##calculate SD for each ion

QCmean<-data.frame(rowMeans(QCtb, dims = 1)) ##calculate mean for each ion

QCrsd<-QCsd/QCmean; names(QCrsd)[1] <- "RSD" ##calculate RSD

QCFinal<-cbind(QCtb,QCrsd) ##add RSD to QCtb dataframe

write.csv(QCFinal,paste0("Troubleshooting/",NegFileName,"_QC.csv")) ##export QCFinal for records

##combine annotations with ISTD normalized data for rsd filtering##

##Add RSD values at end of each row##

rsd<-data.frame(NegCombineAnnot1,ISTD,NegCombineAnnot2, QCrsd)

##keep peaks with RSD<0.3 or 30%##

##threshold <- 0.3 ##set threshold

##Trim data by the RSD column which is the last column##

ISTDTrimmed<-subset(rsd, rsd[,ncol(rsd)] < QCTol)

ISTDTrimmed = ISTDTrimmed[, -ncol(ISTDTrimmed)] ##remove RSD column

ISTDTrimmed[is.na(ISTDTrimmed)] <- "" ##remove NAs

write.csv(ISTDTrimmed,paste0("Troubleshooting/",NegFileName,"_QCTrimmed.csv")) ##export for records

}else{

ISTDTrimmed<-data.frame(NegCombineAnnot1,ISTD,NegCombineAnnot2)

}

NegData<-ISTDTrimmed ##save as NegData object for later

####PosData###

##Create a subset object of integrated peaks and "mzrt" column using date as a column name pattern#

PosRaw<-Pos[,grep(RunCodePos,colnames(Pos))]

##Create a subset of annotations before peaks and annotations after peaks#

PosCombineAnnot<-PosCombine[,-grep(RunCodePos,colnames(PosCombine))] ##subset without integrated peaks

PosCombineAnnot1<-PosCombineAnnot[,1:(ncol(PosCombineAnnot)-3)]##subset before peaks

PosCombineAnnot2<-PosCombineAnnot[,(ncol(PosCombineAnnot)-2):ncol(PosCombineAnnot)]##subset after peaks

if(ISTDNorm){

##Find ISTD row##

rownames(PosRaw)

##and rename to "ISTD"##

ISTDPosrow<-grep(PosISTD, rownames(PosRaw))

rownames(PosRaw)[ISTDPosrow]<-"ISTD"

##Check rownames to confirm

rownames(PosRaw)

##convert to data matrix##

df1 = as.matrix(PosRaw)

##Divide all rows by ISTD Row (Must find ISTD Row Number)##

ISTDPosMean<-mean(df1["ISTD",], dims = 1)

df1["ISTD",]<-sapply(df1["ISTD",],function(x) x/ISTDPosMean)

ISTDPos<-t(t(df1)/df1["ISTD",])

PosFileName<-paste0(filename,"_Pos")

##export ISTD normalized data for records

write.csv(ISTD,paste0("Troubleshooting/",PosFileName,"_ISTD.csv"))

}else{

PosFileName<-paste0(filename,"_Pos")

ISTDPos<-PosRaw

}

##Determine max peak intensity in each row##

maxint<-apply(ISTDPos, 1, max)

PosCombineAnnot2<-data.frame(maxint,PosCombineAnnot2)

####Step 3:Evaluate QC Variability####

if(QCFilter){

QC<-t(ISTDPos) ##tranpose ISTD dataframe

QCtb <- QC[grep("*Pool*",rownames(QC)),] ##subset only Pool injections

QCtb<-t(QCtb) ##transpose again

QCsd<-data.frame(apply(QCtb, 1, sd)); names(QCsd)[1] <- "SD" ##calculate SD for each ion

QCmean<-data.frame(rowMeans(QCtb, dims = 1)) ##calculate mean for each ion

QCrsd<-QCsd/QCmean; names(QCrsd)[1] <- "RSD" ##calculate RSD

QCFinal<-cbind(QCtb,QCrsd) ##add RSD to QCtb dataframe

write.csv(QCFinal,paste0("Troubleshooting/",PosFileName,"_QC.csv")) ##export QCFinal for records

##combine annotations with ISTD normalized data for rsd filtering##

##Add RSD values at end of each row##

rsd<-data.frame(PosCombineAnnot1,ISTDPos,PosCombineAnnot2, QCrsd)

##keep peaks with RSD<0.3 or 30%##

##threshold <- 0.3 ##set threshold

##Trim data by the RSD column which is the last column##

ISTDTrimmed<-subset(rsd, rsd[,ncol(rsd)] < QCTol)

ISTDTrimmed = ISTDTrimmed[, -ncol(ISTDTrimmed)] ##remove RSD column

ISTDTrimmed[is.na(ISTDTrimmed)] <- "" ##remove NAs

write.csv(ISTDTrimmed,paste0("Troubleshooting/",PosFileName,"_QCTrimmed.csv")) ##export for records

}else{

ISTDTrimmed<-data.frame(PosCombineAnnot1,ISTDPos,PosCombineAnnot2)

}

PosData<-ISTDTrimmed ##save as PosData object for later

#####Merge Neg and Pos####

##make dataframe of NegIonBoth, maxint for NegData

NegToMergePos<-data.frame(NegData$NegIonBoth.Compound,NegData$maxint); names(NegToMergePos)[1]<-"IonInBoth"

NegToMergePos[NegToMergePos==""]<-NA ##set blank spaces to NA

NegToMergePos<-subset(NegToMergePos,!is.na(IonInBoth)) ##remove NA in "IonInBoth"Column

##make dataframe of NegIonBoth (used to merge with NegData above), PosIonBoth and maxint for PosData

PosToMergeNeg<-data.frame(PosData$NegIonBoth.Compound, PosData$PosIonBoth.Compound,PosData$maxint); names(PosToMergeNeg)[1]<-"IonInBoth"

PosToMergeNeg[PosToMergeNeg==""]<-NA

PosToMergeNeg<-subset(PosToMergeNeg,!is.na(IonInBoth))

NegPosMerge<-merge(NegToMergePos,PosToMergeNeg, by = "IonInBoth", all = FALSE)

##Determine Neg ions that are lower than Pos ions to be removed

theMaxNegtoRemove<-data.frame(subset(NegPosMerge, NegPosMerge$NegData.maxint<NegPosMerge$PosData.maxint))

names(theMaxNegtoRemove)[1]<-"NegIonBoth.Compound"

theMaxNegtoRemove$PosData.PosIonBoth.Compound<-NULL

##Combine NegData and Neg Ions to remove then export to neg folder for records##

NegRemoveRaw<-merge(NegData,theMaxNegtoRemove, by="NegIonBoth.Compound", all.x = TRUE) ##merge Neg and the ions to remove sheets

NegRemoveRaw[is.na(NegRemoveRaw)] <- "" ##replace NAs with space

NegRemoveRaw<-as.data.frame(append(NegRemoveRaw, NegRemoveRaw[1], after = ncol(NegRemoveRaw)-1)) ##move column merged (column 1) to end of data sheet

NegRemoveRaw<-NegRemoveRaw[,-1] ## delete first column

##You can use this exported spreasheet to check if the correct ions were removed for the pos/neg merge because it contains all of them

write.csv(NegRemoveRaw,paste0("Troubleshooting/",NegFileName,"_ALLwithNegToRemove.csv"))

write.csv(theMaxNegtoRemove,paste0("Troubleshooting/",NegFileName,"_NegToRemove.csv"))

NegFinal<-NegData[!NegData$NegIonBoth.Compound %in% theMaxNegtoRemove$NegIonBoth.Compound,] ##remove pcgroups in MaxNegtoRemove from Neg

##Determine Pos ions that are lower than Neg ions to be removed

theMaxPostoRemove<-data.frame(subset(NegPosMerge, NegPosMerge$NegData.maxint>NegPosMerge$PosData.maxint))

names(theMaxPostoRemove)[3]<-"PosIonBoth.Compound"

##Combine PosDataFiltered and Pos Ions to remove then export to Pos folder for records##

PosRemoveRaw<-merge(PosData,theMaxPostoRemove, by="PosIonBoth.Compound", all.x = TRUE)

PosRemoveRaw[is.na(PosRemoveRaw)] <- "" ##replace NAs with space

PosRemoveRaw<-as.data.frame(append(PosRemoveRaw, PosRemoveRaw[1], after = ncol(PosRemoveRaw)-1))

PosRemoveRaw<-PosRemoveRaw[,-1]

write.csv(PosRemoveRaw,paste0("Troubleshooting/",NegFileName,"_ALLwithPosToRemove.csv"))

write.csv(theMaxPostoRemove,paste0("Troubleshooting/",NegFileName,"_PosToRemove.csv"))

PosFinal<-PosData[!PosData$PosIonBoth.Compound %in% theMaxPostoRemove$PosIonBoth.Compound,]

PosFinal[is.na(PosFinal)] <- ""

####Step 5: Create EZInfo sheet for Pos and Neg Combined#####

NegEZInfo<-t(data.frame(NegFinal[,grep(RunCodeNeg,colnames(NegFinal))]))

PosORNeg<-rep("Neg",ncol(NegEZInfo))

NegEZInfo<-rbind(t(NegFinal[,1:3]),PosORNeg,NegEZInfo)

NegEZInfo<-data.frame(rownames(NegEZInfo),NegEZInfo); rownames(NegEZInfo)<-NULL; colnames(NegEZInfo)[1]<-"SampleName"

PosEZInfo<-t(data.frame(PosFinal[,grep(RunCodePos,colnames(PosFinal))]))

PosORNeg<-rep("Pos",ncol(PosEZInfo))

PosEZInfo<-rbind(t(PosFinal[,1:3]),PosORNeg,PosEZInfo)

PosEZInfo<-data.frame(rownames(PosEZInfo),PosEZInfo); rownames(PosEZInfo)<-NULL; colnames(PosEZInfo)[1]<-"SampleName"

##Check that samples are in same order for Pos and Neg Mode

SampleNames<-data.frame(NegEZInfo$SampleName,PosEZInfo$SampleName)

write.csv(SampleNames,paste0("Troubleshooting/",NegFileName,"_SampleNameLineUp.csv"))

##Delete Pos mode SampleName column and combine Neg and Pose dataframes

PosEZInfo$SampleName<-NULL

NegPosCombineEZInfo<-data.frame(NegEZInfo,PosEZInfo)

write.csv(NegPosCombineEZInfo,paste0(filename,"_SIMCA.csv"), row.names = FALSE)

return(NegPosCombineEZInfo)

}else{

write.csv(NegIonBoth,"NegDoesNotMatchPos_NegIons.csv")

write.csv(PosIonBoth,"PosDoesNotMatchNeg_PosIons.csv")

}

}

setwd("M:/Velenosi/RScripts/")

save(ProgenCombineNegPos,file="ProgenCombineNegPosFunctionFinal.Rdata")

##String split to obtain group labels

EZInfoGroups<-strsplit(as.character(NegPosCombineEZInfo$SampleName),"_")##string split

EZInfoGroups[1]<-NULL##remove PosORNeg

EZInfoGroups<-paste(sapply(EZInfoGroups, "[[",1), sapply(EZInfoGroups, "[[",2), sep = "_") ##obtain group labels

EZInfoGroups<-gsub("_CB151022XG","",EZInfoGroups) ##replace Neg with Pool

EZInfoGroups<-append("",EZInfoGroups)##insert blank cell in list for PosOrNeg

##Add SampleGroup labels##

NegPosCombineEZInfo<-data.frame(append(NegPosCombineEZInfo, list(SampleGroups=EZInfoGroups), after = 1)); rownames(NegPosCombineEZInfo)<-NULL

##Remove date and Mode from SampleName Column

NegPosCombineEZInfo$SampleName<-gsub("CB151022XG2_","",NegPosCombineEZInfo$SampleName)

NegPosCombineEZInfo$SampleName<-gsub("_Neg","",NegPosCombineEZInfo$SampleName)

##Export EZinfo csv

write.csv(NegPosCombineEZInfo, "RPLC_CombinedMode_EZinfo_Final.csv", row.names = FALSE)

load("RPLC_CombinedFINAL.RData")
